# Progression of Voice and Speech Impairment in the Course of Parkinson's Disease: A Longitudinal Study

**DOI:** 10.1155/2013/389195

**Published:** 2013-12-10

**Authors:** S. Skodda, W. Grönheit, N. Mancinelli, U. Schlegel

**Affiliations:** Department of Neurology, Knappschaftskrankenhaus, Ruhr-University of Bochum, In der Schornau 23-25, 44892 Bochum, Germany

## Abstract

Impairment of voice and speech occurs in the majority of patients in the course of Parkinson's disease (PD). The aim of the current study was to survey the changes of voice and speech performance in the individual patients over time. 80 patients with PD and 60 healthy speakers were tested and retested after at least 12 months (average time interval: 32.5 months). Participants had to read a given text which was digitally recorded as a source for the perceptual and acoustic analysis. Stage of the disease and global motor impairment were rated according to the accepted scales. As a result, abnormalities of voice and speech were already present in mildly affected patients and there were significant deteriorations of quality of voice and articulatory velocity and precision between baseline and followup examination which showed no correlation with the time interval between the visits. Summarized, voice, and speech performance were found to further deteriorate in the individual patient in the course of time although global motor impairment was widely stable which might be a hint for nondopaminergic mechanisms of progression of dysarthrophonia. Further investigations are warranted to get a better insight into the dynamics of the progression of voice and speech impairment in PD as a precondition for the development of therapeutic approaches.

## 1. Introduction 

Voice and speech impairment (also called “dysarthrophonia”) is a typical symptom of Parkinson's disease (PD) and occurs in the majority of patients in the course of the illness [1–3]. The progressive loss of the ability to communicate is considered to be an important source of disability in patients with PD [3–6]. The typical pattern of hypokinetic dysarthria is characterized by a breathy or hoarse voice, reduced loudness and restricted pitch variability (monopitch and monoloudness), imprecise articulation and abnormalities of speech rate, and pause ratio (e.g., [7–9]). These multidimensional abnormalities of voice and speech have traditionally been attributed to the dopaminergic deficit manifesting in hypokinesia and rigidity of the laryngeal muscles [10, 11]. Indeed, there is some evidence for an amelioration of at least some single speech dimensions such as pitch and loudness variability under dopaminergic treatment (e.g., [12, 13]). However, other studies have failed to demonstrate a clear causal relationship between dopaminergic dysfunction and overall speech performance (e.g., [14]); therefore, it had been suggested that alterations of voice and speech in PD might be at least partly due to nondopaminergic mechanisms with additional alteration of internal cueing, sensorimotor gating, scaling, and timing of speech movements [15–18].

Voice and speech abnormalities have been found to be more severe in the advanced stages of PD [1, 18, 19]; however, data on development and progression of dysarthria in the individual patients are sparse. In a previous investigation of our group, some single acoustic speech parameters as articulation rate and pitch variability in female speakers were found to further decrease in the course of time [20]. In the same vein, vowel articulation was demonstrated to deteriorate between baseline and a followup examination performed at least after 12 months in a group of 67 patients with PD [21]. However, these studies were reduced to the monitoring of some separate speech parameters based on acoustic analyses without including overall speech performance and intelligibility.

Based upon these previous findings, the aim of the current study was to examine a variety of measures of voice and speech derived from acoustic and perceptual analyses in an even larger group of patients with PD in the course of time. We also intended to relate the observed changes of voice and speech variables to overall motor performance and the stage of the disease according to Hoehn-Yahr stages. Furthermore, the PD group was compared with a control group of similar age and gender distribution which was also tested and retested after a similar time interval to account for possible effects of aging alone. According to our previous findings, our parameters of speech chosen for the monitoring over time were hypothesized to be independent of dopaminergic regulation. Therefore, in this present study, we intended to keep patients' overall motor performance widely stable over time by individually adjusting the dopaminergic medication when necessary in order to prove if speech performance was deteriorating nonetheless which could serve as a hint for the nondopaminergic control of at least certain aspects of voice and speech in PD.

## 2. Patients and Methods

From 2002 to 2012, 80 patients (48 male) with *idiopathic Parkinson's disease (PD)* were recruited for this study. The diagnosis of PD was based upon clinical criteria according to the UK Parkinson's Disease Society Brain Bank Criteria. Patients' age on first examination ranged from 40 to 80 years (mean: 66.28/median: 67/SD: 8.11). PD had been diagnosed from 1 to 20 years prior to the first examination (mean: 6.09/median 5/SD: 4.63). Time between first and second examination ranged from 12 to 88 months (mean: 32.53/median: 29/SD: 19.53). On both visits, each patient underwent a neurological examination, according to Unified Parkinson's Disease Rating Scale Motor Score/UPDRS III. Item 18 of the UPDRS Motor Score “speech” was taken for global perceptual description of patients' speech. The stage of the disease at baseline was determined according to the Hoehn-Yahr scale in the medical “on”-state (mean: 2.16/median: 2/SD: 0.58/range 1 to 4). Accordingly, patients were classified as “mildly affected” (Hoehn-Yahr stages 1 and 1.5, *n* = 19), “moderately affected” (Hoehn-Yahr 2 and 2.5, *n* = 49), and “severely affected” (Hoehn-Yahr 3 and 4, *n* = 12). At the followup visit, and none of the participants had passed over to the more affected group.

A subgroup of 46 patients and 17 controls had participated in a previous study on speech performance [20, 21]. At the time of the examination, patients were on stable dopaminergic medication since at least 4 weeks prior to the examination. Speech and motor examinations were performed 60 to 90 minutes after the morning dose of medication to ensure the “on”-state. None of the patients experienced orofacial or abdominothoracic peak-dose dyskinesia during the examination. Medication with anticholinergics, cholinesterase inhibitors and atypical neuroleptics, and severe dementia (MMSE < 25 pts.) were the exclusion criteria.

As* control group* we tested and retested 60 age-matched healthy persons (mean age 66.87 years/median 67.5 years/SD: 7.10/range 55 to 80 years; 30 male) which were retested after a mean time period of 25.39 months (median 21/SD 6.86/range 12 to 40 months). Participants' characteristics are shown in Table 1.

All participants were native German speakers. None of the participants suffered from relevant hearing impairment as assessed by a hearing screening test (exposition to test sounds prior to the definite examination).

For the speech test, each participant had to read a given text composed of four phonetically balanced sentences; furthermore, participants had to produce the vowel /a/ as long as possible. Speech samples were digitally recorded using a commercial audio software (Steinberg WaveLab/Steinberg Media Technologies GmbH, Hamburg, Germany) and a headset microphone with a defined mouth to microphone distance of 3 cm. Speech records of the reading task were perceptually analyzed independently by two examiners (S. Skodda and W. Grönheit), who were blinded for the speakers' condition, according to a four-dimensional scoring system which is used for the description of Parkinsonian dysarthria in our clinic (Table 2). Additionally, we applied an ordinal scale using verbal depiction of overall speech intelligibility with four classifications (“good,” “fair,” “moderate,” poor”) which has been previously found to exhibit good correlations to the more often used visual analogue scale/VAS (e.g., [22–24]).

Interrater reliability was high with *w* = 0.924; in cases of divergent ratings, the higher score was chosen. Additionally, acoustic analysis of speech was performed for several speech parameters for the objective description of voice, articulation, fluency, and prosody by the use of PRAAT [25] (Table 3). Jitter, shimmer, and noise to harmonics ratio as measures of voice quality were based upon the analysis of sustained phonation (e.g., [26]). Mean fundamental frequency (mean *F*_0_) of the reading task was taken as measure of phonation. Description of intonation variability was based upon standard deviation of the fundamental frequency (*F*_0_ SD). Analysis of speech rate was performed by measuring the length of each syllable and each pause, respectively, based on the oscillographic sound pressure signal. Besides the conventional speech rate variables as net speech rate (NSR) and pause ratio (PR%), we additionally defined the percental ratio of pauses within polysyllabic words (Pinw%), which can be taken as a measure of precision of stop consonant articulation [27]. Description of vowel articulation was based upon the recently established vowel articulation index/VAI which is a surrogate parameter of the first and second formant frequencies (*F*_1_ and *F*_2_) of the three corner vowels /*α*/, /i/, and /u/ [28, 29]. Since mean *F*_0_, *F*_0_ SD, and VAI are related to the speaker's pitch of voice, the comparison of these parameters between PD patients and controls was performed separately for both genders.

Winstat^©^ (Bad Krotzingen/Germany) was used for statistical analyses. Paired and unpaired *t*-test were performed for intragroup comparison (*t*_0_ versus *t*_1_) and comparison between groups, since the variables were largely normally distributed (Shapiro-Wilk test). For the calculation of interrater reliability, Kendall's coefficient of concordance was used. Pearson correlation and Spearman rank test were used to perform correlation analyses. Effect sizes were measured with Cohen's *d* with *d* > 0.5 indicating a medium and *d* > 0.8 indicating a large effect. Due to the exploratory nature of the study, no adjustments for multiple comparisons were made, and the level of significance was set at *P* < 0.05.

Our study was in compliance with the Helsinki Declaration and had been approved by the local Ethics Committees. Written informed consent was obtained from each participant.

## 3. Results

### 3.1. Correlations between Perceptual Scores and Acoustic Analysis

In the PD group at baseline/*t*_0_, the UPDRS speech item showed significant correlations with the perceptual speech score (“voice”: *r* = 0.406, *P* < 0.0001; “articulation”: *r* = 0.364, *P* = 0.0004; “fluency”: *r* = 0.472, *P* < 0.0001; “prosody”: *r* = 0.383, *P* = 0.0002). The perceptual sum score was highly correlated to the overall intelligibility score as well (*P* < 0.0001).

On the other hand, perceptual ratings were correlated to some of the measures of acoustic analysis, namely, NSR and the perceptually rated “fluency” score (*r* = 0.296, *P* = 0.004) and Pinw% and all perceptual subscores (“voice”: *r* = −0.380, *P* = 0.0003; “articulation”: *r* = −0.480, *P* < 0.0001; “fluency”: *r* = −0.322, *P* = 0.002; “prosody”: *r* = −0.283, *P* = 0.006). Furthermore, in gender-based calculation, correlations were found between mean *F*_0_ and “voice” (*r* = 0.284, *P* = 0.025) and *F*_0_ SD and “articulation” (*r* = −0.438, *P* = 0.001) and “prosody” (*r* = −0.486, *P* = 0.0002) in the male PD group; however, in the female PD group, there were only correlations between *F*_0_ SD and “prosody” (*r* = −0.340, *P* = 0.028).

### 3.2. Comparison between PD Group and Control Group at Baseline/*t*_0_

Based upon the perceptual score, all speech modalities were significantly worse in the PD group than in the control group at baseline (*P* < 0.0001). According to the acoustic analysis, values for shimmer and nhR, but not for jitter, were significantly higher in the PD group (*P* < 0.0001); mean *F*_0_ was elevated in males (*P* = 0.035), but not in female speakers with PD. Pinw% was found to be significantly reduced (*P* < 0.0001), whereas VAI showed a significant reduction only in female Parkinsonian speakers (*P* = 0.026/for male speakers: *P* = 0.094). Concerning speech rate, there was a reduction of PR% (*P* = 0.003) and a tendency to increased NSR (*P* = 0.085). Furthermore, there was a tendency to reduced *F*_0_ SD in male PD speakers (*P* = 0.078) and significantly reduced *F*_0_ SD in female PD speakers (*P* < 0.0001).

### 3.3. Comparison of Speech Parameters at Baseline/*t*_0_ and Followup/*t*_1_

In the PD group, overall speech performance according to UPDRS speech item as well as the subscores of the perceptual speech score showed a significant increase indicating a deterioration of all speech modalities and of overall intelligibility over time (*P* < 0.0001). Concerning the measures of acoustic analysis, significant differences were found for shimmer, nhR, NSR, Pinw%, and VAI.

No such differences were seen between *t*_0_ and *t*_1_ in the control group (numerical data are listed in Table 1).

**Figure 1:**
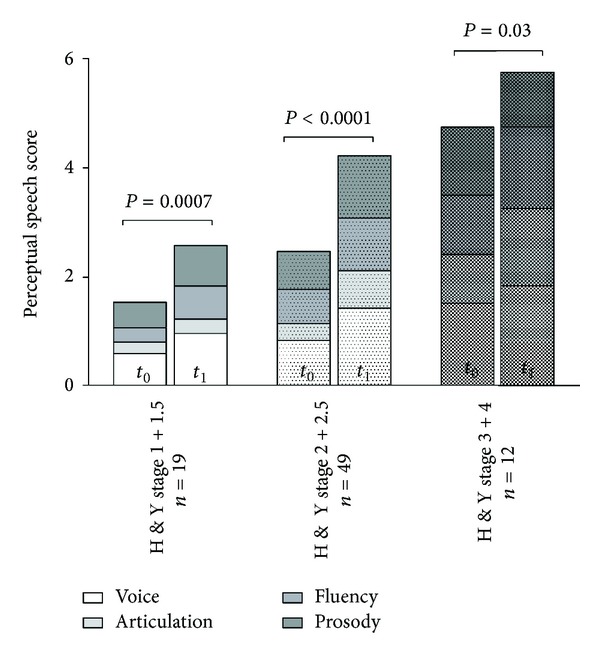
Average values of the perceptual speech scores at baseline (left column) and at followup (right column), subdivided according to the stage of disease.

There were no correlations between the intelligibility score and the time passed between the two examinations (*P* = 0.264) and only weak correlations between the time interval and the changes of the perceptual sum score (*r* = 0.300, *P* = 0.02). However, significant correlations were found between the baseline Hoehn-Yahr stage and the items of the perceptual speech score (voice: *r* = 0.429, *P* < 0.0001; articulation: *r* = 0.419, *P* < 0.0001; fluency: *r* = 0.396, *P* = 0.0001; prosody: *r* = 0.331, *P* = 0.001). Correlations to the baseline UPDRS motor score were found to be weaker, but still significant (voice: *r* = 0.270, *P* = 0.008; articulation: not significant; fluency: *r* = 0.474, *P* < 0.0001; prosody: *r* = 0.239, *P* = 0.016). The relationship between degree of perceptually rated voice and speech impairment and Hoehn-Yahr stages is displayed in Figure 1. Concerning the acoustic analysis, correlations were seen between baseline Hoehn-Yahr stages (but not UPDRS motor score) and PR% and Pinw% at baseline (*r* = 0.355, *P* < 0.001 and *r* = −0.447, *P* < 0.0001) and at followup (*r* = 0.355, *P* < 0.001 and *r* = −0.424, *P* < 0.0001).

In the PD group, *n* = 21 patients (15 male) showed a deterioration of overall speech intelligibility from “good” or “fair” (0 or 1) to “moderate” or “poor” (2 or 3) intelligibility indicating the crossing of a boundary of probable clinical relevance. This subgroup was characterized by higher Hoehn-Yahr stages (2.52 ± 0.43 versus 2.03 ± 0.58; *P* = 0.0007), higher UPDRS speech item scores (1.57 ± 0.51 versus 0.80 ± 0.61; *P* < 0.0001), and a tendency to higher overall UPDRS scores (24.25 ± 11.21 versus 18.92 ± 10.67; *P* = 0.060) at baseline. No significant differences were seen concerning age, disease duration at baseline, and the time interval between the examinations.

**Figure 2:**
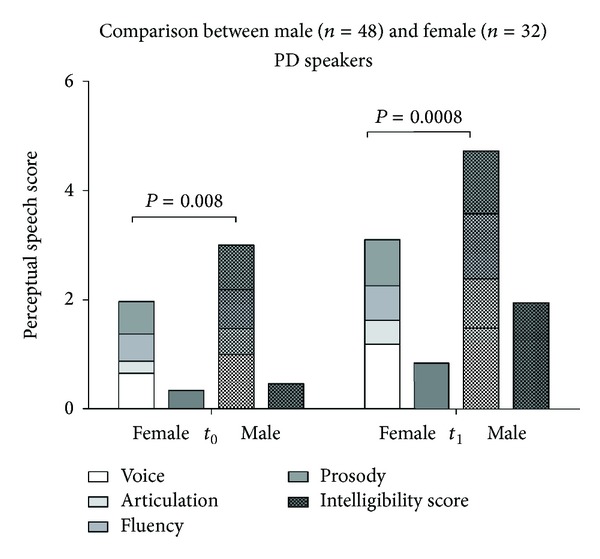
Average values of the perceptual speech scores and the intelligibility score at baseline in female and male speakers at baseline (left column) and at followup (right column).

Furthermore, there were gender-related differences concerning the perceptually rated speech dimensions with poorer performance of the male PD patients which, however, was not mirrored by the overall speech intelligibility (see Figure 2).

## 4. Discussion

This study aimed to survey the development of different measures of voice and speech performance in the clinical course of PD. Patients' speech performance was compared to a longitudinal speech performance of an age- and gender-matched control group in order to disclose normal ageing changes. In the PD group, the perceptual analysis revealed a significant deterioration of all the monitored speech dimensions which could in general be found independent of the stage of the disease at baseline as an indication that abnormalities of voice and speech can already occur in the early stages of PD and continue to worsen in the course of disease progression. Accordingly, in one large previous cross-sectional investigation based upon perceptual analysis, abnormalities of voice were found to be already present in patients with only mild overall motor impairment, and additional decline of articulation and fluency appeared in the more advanced stages of the disease [1]. However, since the majority of patients in our study were in the rather advanced stages of disease at baseline (mean disease duration about six years, average Hoehn-Yahr stage 2.16) and the followup intervals lay within a wide range, our results cannot answer the question if speech deterioration occurs continuously or rather stepwise and if the different speech modalities show a similar pattern of decline in the course of time.

Nonetheless, we were able to document a progression of voice and speech impairment not only based upon perceptual judgment, but also substantiated by objective acoustic measures with a significant increase of shimmer and noise to harmonics ratio as measures of voice quality and a reduction of speech rate, intraword pauses, and the vowel articulation index as indication for a deterioration of articulation and fluency which is in line with previous investigations of our group, however, performed in a smaller group of patients [20, 21]. The combination of perceptual and acoustic analysis of voice and speech as performed in the current study seemed to be appropriate to complimentarily obtain clinical surrogate measures of the different modalities as voice, articulation, fluency and prosody, and objective measures of individual variables in which changes could be too subtle to be detected by perceptual judgment only. In the same vein, elaborate telemetric analyses of a variety of different speech parameters have been successfully used to predict the disease severity in a large number of 82 patients with PD, and another investigation based upon acoustic analysis revealed preclinical abnormalities of voice and prosody in a group of 23 drug naïve patients with early PD [30, 31].

In our study, UPDRS motor score was held widely stable over time (obviously due to an interim adaptation of the dopaminergic medication); hence, the observed changes seem to be independent of global motor function. These findings give reason to the hypothesis that voice and speech impairment could be the result of an escalation of axial dysfunction too subtle to be mirrored by global UPDRS motor score, but rather captured by the Hoehn-Yahr score which could explain its close correlation with the perceptual ratings and some of the acoustic measures (namely, PR% and Pinw%) as well. Alternatively, alterations of speech parameters could be completely independent of motor performance that may be based upon nondopaminergic mechanisms, as it is partly supported by the inconclusive findings concerning the behaviour of speech under levodopa admission [13, 14, 16]. On the other hand, although the examination of patients under their individual therapeutic regimen displays the “naturalistic” clinical situation, our results do not allow any insight into the natural development of voice and speech in the unmedicated course of PD. Furthermore, any negative influence of the dopaminergic medication at least on some distinctive speech parameters cannot be excluded.

For our investigation, we used a reading passage consisting of four phonetically balanced sentences in order to obtain comparable data for the acoustic analysis and to make sure that the perceptual rating was not confounded by, for example, the speakers' spontaneity and eloquence as it could be in an arbitrary monologue. However, it is well known from the literature that the kind of speech task can influence speech performance in patients with movement disorders and in healthy speakers as well. For example, pitch variability has been proven to be higher in reading or deliberately clear speech than in conversation (e.g., [32, 33]). However, since reading an unfamiliar text in a laboratory setting can be assumed to require a certain level of attention and alertness, participants' speech performance should presumably mirror the best remaining speech capacity in the bounds of the illness.

Interestingly, there were differences concerning the perceptual speech ratings between male and female PD speakers which had been previously reported concerning some distinctive measures of acoustic speech analysis [34] and, however, were not mirrored by differences of overall speech intelligibility in our investigation and previous studies (e.g., [24]).

Admittedly, the current study has some methodical limitations which are mainly induced by our attempt to combine acoustic and perceptual measurements of speech in order to achieve a complete description of all the different dimensions of Parkinsonian dysarthria. However, measures of the acoustic analysis showed only weak correlations with perceptual ratings, and no single simple acoustic measure was found to be an adequate surrogate parameter to mirror overall speech intelligibility. Therefore, clinical impact of speech impairment in PD cannot be captured by acoustic analyses alone and should also be combined with the disability or functional changes perceived by the patients themselves and/or their families and caregivers which has not been performed in our study. Furthermore, our perceptual speech analysis was not based upon an extensive assessment executed by a speech therapist but instead was independently performed by two neurologists experienced with the care and treatment of patients with PD using a rather simple four-dimensional rating score. Obviously, this rating score might seem to be questionable because it still lacks the prove of sensitivity, specificity, and reliability in a much larger cohort of patients with PD, but it has at least been found to be easily applicable in the clinical setting with good interrater reliability and plausible correlations to the accordant measures of the acoustic analysis. In addition, we used a simple rating score of overall speech intelligibility which has been found to be sufficiently valid and reliable in the clinical setting in order to fill the gap between the assessment of impaired single speech dimensions and overall speech performance. As a result, overall speech intelligibility also showed a significant worsening between the first and the second examinations and a subgroup of *n* = 21 patients who deteriorated significantly (reaching the level “moderate or poor” intelligibility) which can be supposed to be relevant to the demands of communication. Interestingly, this subgroup was characterized by higher baseline Hoehn-Yahr scores and UPDRS speech items values. As a consequence of these findings, PD patients with already mildly impaired speech performance and Hoehn-Yahr stages of 2.0 and higher seem to be on special risk to develop a substantial decline of speech performance in the near future and should be monitored accordantly.

Summarized, the current study provided preliminary data about the development of voice and speech impairment in a considerable cohort of patients with PD which—as far as we know—has not been performed before. The interpretation of the results is undoubtedly limited by some methodologic limitations mainly due to the uncontrolled followup intervals between the examinations, the implementation of a still unvalidated perceptual speech score, and the lack of assessment of self-perceived communication deficits. Nonetheless, voice and speech deterioration was observed over time although patients were under dopaminergic medication optimized for best motor outcome (and therefore mirroring the patients' typical clinical situation) which justifies the hypothesis of nondopaminergic or special dopaminergic mechanisms responsible for Parkinsonian dysarthrophonia. Unfortunately, the therapeutic approaches for an amelioration of speech performance in PD are still disappointing, and the Lee Silverman Voice Treatment/LSVT which is considered as the most effective speech and therapy, so far, has its limitations mostly by insufficient availability and prescription. Thus, the obvious need of specific and successful speech therapies call for a better understanding of the pathomechanisms and the time course of impairment and further deterioration of voice, articulation, fluency, and prosody. Therefore, future longitudinal studies on voice and speech are warranted, ideally with a baseline examination as soon as the first motor signs of PD are noticeable and with defined followup intervals performed regularly in the course of disease progression.

Our study has provided some first insight into some aspects of the longitudinal development of different speech dimensions and overall speech performance in PD; however, further work is required to establish appropriate methods of speech investigations which produce objective data, fulfil the demands of validity and reproducibility, time and cost effectiveness, and mirror best the functional disability of patients.

## Figures and Tables

**Table 1:** Participants' characteristics and results.

	PD group (*n* = 80; 48 male)						Control group (*n* = 60; 30 male)				
	*t*_0_		*t*_1_				*t*_0_		*t*_1_		
					*t*_0_ versus *t*_1_						*t*_0_ versus *t*_1_
	Mean	S.D.	Mean	S.D.			Mean	S.D.	Mean	S.D.	
Age	66.28	8.11					66.87	7.10			
Disease duration (months)	73.10	55.54	105.63	57.15							
*t*_0_ − *t*_1_ (months)			32.53	19.53					25.39	7.08	
Hoehn&Yahr_*t*_0__	2.16	0.58									
UPDRS III	20.16	10.96	19.58	8.29	n.s.						
UPDRS speech	1.00	0.68	1.39	0.86	*P* < 0.0001						

Perceptual rating					*t*_0_ versus *t*_1_	C *d*					
Intelligibility score	0.41	0.57	0.96	0.92	*P* < 0.0001	0.72	0.08	0.28	0.13	0.34	n.s.
Perceptual score	2.59	1.82	4.06	2.33	*P* = 0.0001	0.71	0.60	0.79	0.70	0.77	n.s.
Voice	0.86	0.67	1.36	0.70	*P* < 0.0001	0.73	0.30	0.46	0.38	0.49	n.s.
Articulation	0.38	0.54	0.71	0.87	*P* < 0.0001	0.46	0.07	0.25	0.13	0.34	n.s.
Fluency	0.63	0.64	0.96	0.95	*P* < 0.0001	0.41	0.02	0.13	0.03	0.18	n.s.
Prosody	0.73	0.64	1.01	0.57	*P* < 0.0001	0.46	0.22	0.42	0.15	0.36	n.s.

Acoustic analysis					*t*_0_ versus *t*_1_	C *d*					
Jitter	1.263	1.038	1.358	0.956	n.s.	—	1.186	0.777	1.205	0.893	n.s.
Shimmer	6.548	3.914	11.490	7.024	*P* < 0.0001	0.87	5.222	2.114	5.209	2.763	n.s.
nh_ratio	0.051	0.062	0.103	0.105	*P* < 0.0001	0.61	0.038	0.029	0.041	0.031	n.s.
Mean *F*_0_—male	133.33	24.20	131.14	23.88	n.s.	—	124.31	12.71	123.58	13.04	n.s.
Mean *F*_0_—female	186.34	25.04	183.93	31.22	n.s.	—	186.29	15.19	188.62	17.21	n.s.
*F*_0_ SD—male	16.90	5.24	15.94	5.93	n.s.	—	19.27	6.40	19.02	6.59	n.s.
*F*_0_ SD—female	23.00	6.55	22.21	0.30	n.s.	—	34.11	8.06	33.91	8.55	n.s.
NSR	5.36	0.75	5.19	0.68	*P* = 0.0003	0.24	5.18	0.48	5.16	0.50	n.s.
PR%	14.65	5.35	15.30	7.05	n.s.	—	17.19	4.38	17.84	4.33	n.s.
Pinw%	21.24	11.15	18.46	10.55	*P* = 0.016	0.26	29.96	9.98	28.62	10.21	n.s.
VAI—male	0.740	0.081	0.677	0.069	*P* < 0.0001	0.84	0.767	0.058	0.759	0.063	n.s.
VAI—female	0.837	0.066	0.792	0.052	*P* < 0.0001	0.76	0.874	0.062	0.861	0.069	n.s.

n.s.: not significant; S.D.: standard deviation; C *d*: Cohen's *d*/effect size.

*t*_0_ versus *t*_1_: comparison between baseline and follow-up/paired *t*-test.

**Table 2:** Perceptual speech score and intelligibility score.

Speech modality		Definition
Voice	0	Normal
	1	Voice quality slightly hoarse, slightly reduced loudness, intermittently present
	2	Voice quality hoarse or tremulous, slightly reduced loudness, continuously present
	3	Voice quality hoarse or tremulous, markedly reduced loudness
	4	Marked reduction of voice quality, whispery or scratchy voice

Articulation	0	Normal articulation
	1	Slightly reduced articulatory accuracy, intermittently present
	2	Slightly reduced articulatory accuracy, continuously present
	3	Markedly reduced articulatory accuracy, slightly reduced intelligibility
	4	Markedly reduced intelligibility

Tempo/fluency	0	Normal speech tempo and distribution of speech pauses
	1	Slightly reduced or accelerated speech tempo, intermittently present
	2	Rushes of speech and prolonged pauses, not very pronounced or only intermittently present; or slightly reduced speech tempo
	3	Rushes of speech and prolonged pauses, very pronounced, or continuously present; or markedly reduced speech tempo
	4	Palilalia

Prosody	0	Normal pitch variability
	1	Slightly monotone
	2	Extremely monotone

Intelligibility score	0	Good intelligibility
	1	Fair intelligibility
	2	Moderately impaired intelligibility
	3	Poor intelligibility

**Table 3:** Abbreviations and definitions of the speech parameters.

Speech modality	Parameter	Definition
Voice	Jitter (measure of microperturbations of frequency)	Average absolute difference between consecutive differences between consecutive periods, divided by the average period
	Shimmer (measure of microperturbations of amplitude)	Average absolute difference between consecutive differences between the amplitude of consecutive periods
	Noise to harmonics ratio (nhR)	Automatic comparison of harmonic (periodically recurring) and inharmonic sound fractions
	Mean *F*_0_	Average fundamental frequency *F*_0_ calculated for the entire reading task

Articulation	Vowel articulation index (VAI)	Comprehensive measure of the “working space” for vowels based upon the extraction of formant frequencies of defined vowels of the reading task according to the formula; VAI = (*F*_2_/i/ + *F*_1_/*α*/) / (*F*_1_/i/ + *F*_1_/u/ + *F*_2_/u/ + *F*_2_/*α*/)
	Percentage of pauses within polysyllabic words (Pinw%)	Percentage of pauses within polysyllabic words of total speech pauses (periods of silence < 10 ms)

Tempo/fluency	Net speech rate (NSR)	Net production of syllables per second based upon the reading task
	Pause ratio (PR%)	Percentage of pause rate based upon the reading task

Prosody	*F*_0_ SD	Standard deviation of fundamental frequencies calculated for the reading task as a measure of pitch variability
